# Inhibition of PDE1-B by Vinpocetine Regulates Microglial Exosomes and Polarization Through Enhancing Autophagic Flux for Neuroprotection Against Ischemic Stroke

**DOI:** 10.3389/fcell.2020.616590

**Published:** 2021-02-04

**Authors:** Jiankun Zang, Yousheng Wu, Xuanlin Su, Tianyuan Zhang, Xionglin Tang, Dan Ma, Yufeng Li, Yanfang Liu, Ze’an Weng, Xuanzhuo Liu, Chi Kwan Tsang, Anding Xu, Dan Lu

**Affiliations:** ^1^Department of Neurology and Stroke Center, The First Affiliated Hospital, Jinan University, Guangzhou, China; ^2^Clinical Neuroscience Institute, The First Affiliated Hospital of Jinan University, Guangzhou, China; ^3^Department of Neurology, The Affiliated Hospital of Youjiang Medical University for Nationalities, Baise, China; ^4^Section of Molecular Computational Biology, Department of Biological Sciences, University of Southern California, Los Angeles, CA, United States

**Keywords:** BV2, OGD, PDE1, vinpocetine, autophagy, exosome

## Abstract

Exosomes contribute to cell–cell communications. Emerging evidence has shown that microglial exosomes may play crucial role in regulation of neuronal functions under ischemic conditions. However, the underlying mechanisms of microglia-derived exosome biosynthesis are largely unknown. Herein, we reported that the microglial PDE1-B expression was progressively elevated in the peri-infarct region after focal middle cerebral artery occlusion. By an oxygen-glucose-deprivation (OGD) ischemic model in cells, we found that inhibition of PDE1-B by vinpocetine in the microglial cells promoted M2 and inhibited M1 phenotype. In addition, knockdown or inhibition of PDE1-B significantly enhanced the autophagic flux in BV2 cells, and vinpocetine-mediated suppression of M1 phenotype was dependent on autophagy in ischemic conditions. Co-culture of BV2 cells and neurons revealed that vinpocetine-treated BV2 cells alleviated OGD-induced neuronal damage, and treatment of BV2 cells with 3-MA abolished the observed effects of vinpocetine. We further demonstrated that ischemia and vinpocetine treatment significantly altered microglial exosome biogenesis and release, which could be taken up by recipient neurons and regulated neuronal damage. Finally, we showed that the isolated exosome *per se* from conditioned BV2 cells is sufficient to regulate cortical neuronal survival *in vivo*. Taken together, these results revealed a novel microglia-neuron interaction mediated by microglia-derived exosomes under ischemic conditions. Our findings further suggest that PDE1-B regulates autophagic flux and exosome biogenesis in microglia which plays a crucial role in neuronal survival under cerebral ischemic conditions.

## Highlights

- PDE1-B expression increases in OGD-conditioned microglial cells in ischemic brain tissues and BV2 cells *in vitro*.
- Vinpocetine suppresses M1 BV2 cell activation and promotes OGD-BV2 survival in an autophagy-dependent manner.
- Inhibition of PDE1-B by vinpocetine enhances the autophagic flux and alters exosome biogenesis in OGD-BV2 cells.
- Vinpocetine treated OGD-BV2 exosomes reverses the OGD-BV2 exosome induced recipient neuronal cell apoptosis and neurite dysfunction.

## Introduction

Phosphodiesterase enzyme (PDE) is known as a calcium- and calmodulin-dependent phosphodiesterase. By catalyzing the hydrolysis of cAMP and cGMP, PDE limits the intracellular levels of cyclic nucleotides and thus regulates the amplitude, duration, and compartmentation of cyclic nucleotide signaling. Because cAMP and cGMP are two critical intracellular secondary messengers that regulate cell signaling pathways, PDE is involved in many cellular processes, including platelet functions (Liu et al., 2014), stabilization of the blood-brain barrier (Kraft et al., 2013; Bieber et al., 2019), and inflammation (Vijayakrishnan et al., 2007). The superfamily of PDE enzymes is classified into 11 groups, including PDE1–PDE11 in mammals (Gulati et al., 2019), and PDE1 is the best characterized (Kakkar et al., 1999). For another, three PDE1 isoforms have been identified (PDE1A, PDE1B, and PDE1C). All the isoforms are expressed within the central nervous system, and PDE1-B is mainly distributed in the cortex (Siuciak et al., 2007). Vinpocetine is a synthetic derivative of the vinca alkaloid vincamine and a specific inhibitor of PDE1 (Zhang Y.S. et al., 2018). Large body of evidence has shown that vinpocetine possesses diverse therapeutic effects on a myriad of diseases, such as osteoporosis (Zhu et al., 2019), age-related macular degeneration (Gao et al., 2019), and liver fibrosis (Essam et al., 2019). In particular, vinpocetine has been reported for its neuroprotective effects on reducing neuroinflammation, improving cerebral blood flow, and synaptic plasticity after ischemic stroke (Sauer et al., 1988; Szilagyi et al., 2005; Vas and Gulyas, 2005; Medina et al., 2006; Krahe et al., 2009; Zhang W. et al., 2016) in rodents and humans. It has been reported that vinpocetine suppresses the release of proinflammatory molecules and proliferation of microglia by inhibiting NF-κB pathway (Zhao et al., 2011; Zhang and Yang, 2014). However, the mechanism of action of vinpocetine in microglia under ischemic stroke condition is not fully understood.

Microglial cells are distributed in the central nervous system and play a critical role in ischemic stroke. Having a similar function to macrophages, microglia recognize and rapidly phagocytose dead cells and regulates homeostasis in the brain. Therefore, they are highly involved in regulation of neuronal functions and survival by several mechanisms including the production of neurotrophic factors, proinflammatory cytokines or extracellular vesicles (Fricker et al., 2018). Normal microglia remain in a resting state, while damaged cells or pathogens stimulate normal microglia to become the activated M2 or M1 microglia which promote the generation of anti-inflammatory or proinflammatory cytokines respectively. In addition, microglial activation is a common feature of many neurodegenerative diseases (Prinz et al., 2011) and ischemic stroke (Ma et al., 2017). Activated microglia induce neuronal dysfunction by inducing apoptosis, excitotoxicity and necrotic death (Block et al., 2007; Brown and Vilalta, 2015) in many neurodegenerative diseases and ischemic stroke (Kim et al., 2015). In addition to inflammatory cytokines, recent findings have revealed that microglia have a strong secretory capacity in releasing exosomes which have been shown to play important roles in regulation of cellular microenvironment during ischemic stroke (Zagrean et al., 2018).

Exosome is one of the cellular microvesicle with diameters of 30–200 nm originating from the endocytic pathway. Recent evidence has revealed that microglia-derived exosomes play an important role in cell–cell communication regulating brain cell survival after brain damage. It has been reported that microglial exosomes are also involved in crosstalk among innate immune cells, microglial training, and neuroinflammation (Lemaire et al., 2019; Song Y. et al., 2019; Hou et al., 2020). It becomes increasingly clear that microglial exosomes contain various cellular metabolites dependent on the status of microglia and they can have diverse effects on the recipient cells. For example, M2 microglia-derived exosomes protect the mouse brain from ischemia-reperfusion injury by exosomal miR-124 (Song Y. et al., 2019). In the context of brain tumors, exosomes could transfer transcripts of several inflammation-related genes from one microglial cell to another dysfunctional microglial cell (Grimaldi et al., 2019). In addition, it has been reported that inhibition of microglial exosome secretion or altering microglial cell states could block the transmission of alpha-synuclein and influence PD progression (Xia et al., 2019). A recent study has showed that miR-124 in the microglial exosomes secreted after ischemic-reperfusion injury has neuroprotective effect (Zagrean et al., 2018). Interestingly, another report also showed that miR-124-3p could alleviates neurodegeneration and improves cognitive function after traumatic brain injury (Ge et al., 2020). These studies indicate that microglial exosomes play novel roles during brain damages. However, the underlying mechanism by which microglia produce and release their exosomes after ischemic stroke remains poorly understood.

In this study, we investigated the mechanism of exosomes released from microglia and their functional role in regulation of neurite morphology and neuronal survival by different ischemic stroke models. We found that PDE1-B plays a role in upregulation of autophagic flux and exosome release after ischemia in cerebral cortex. In addition, PDE1-B inhibition by vinpocetine treatment significantly enhances exosome released from microglia and protects neuronal cells against ischemic damage.

## Results

### Elevated Expression of PDE1-B in Microglial Cells in Ischemic Brain Tissue

**FIGURE 1:**
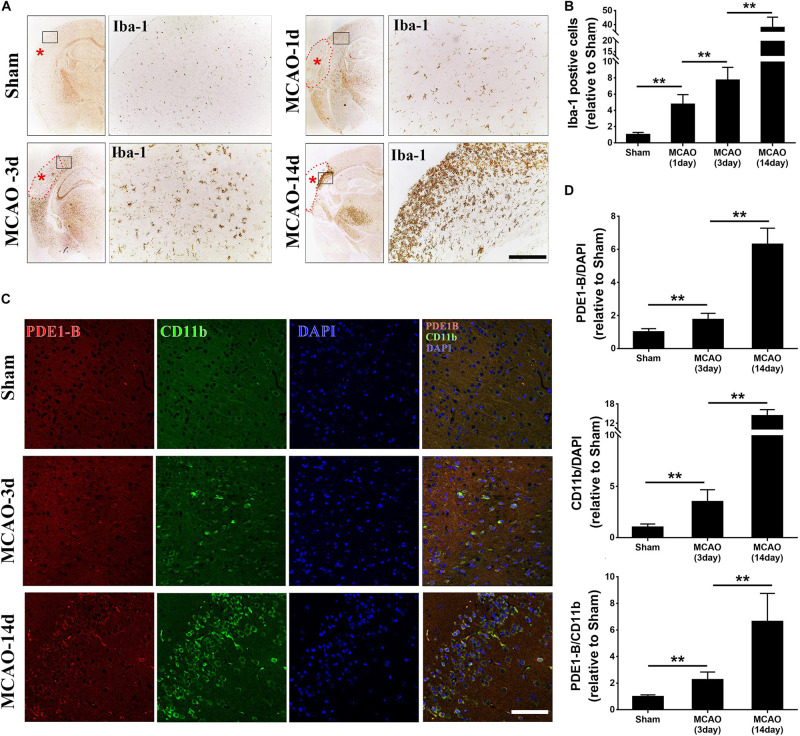
Microglial activation and PDE1-B expression are significantly increased in the ischemic brain tissue. **(A)** Adult C57/B6 mice were subjected to sham or focal MCAO surgery and brain sections were collected after 1, 3, and 14 days. Microglial cells were visualized using Iba-1 staining by immunohistochemical microscopy. The dotted line indicates the infarct area. Asterisk indicates the site of MCA occlusion. The boxed areas indicate the peri-infarct regions, which are magnified for better visualization (right panels). **(B)** Quantification data are presented as mean ± SD. **p < 0.01, *N* = 3 mice per group, scale bar = 200 μm. Representative immunofluorescence images **(C)** and quantifications **(D)** of PDE1-B positive M1 microglia (CD11b) in the peri-infarct cortex of mice 3 days and 14 days after MCAO. Nucleus were visualized by DAPI staining. **p < 0.01, *N* = 3 mice per group, scale bar = 100 μm.

To explore the role of PDE1-B in microglia after ischemic stroke, we firstly used the intraluminal suture-mediated transient and permanent MCAO models in mice. However, the high mortality rate of the transient MCAO model prevented us from performing the time course experiments in this study. Thus, we used the distal permanent MCAO model as the survival rate was much higher. We compared the distribution of microglia and their PDE1-B expression. As expected, the Iba-1-positive microglial cells in the peri-infarct ischemic region were significantly increased in the first day and peaked at the 14th day after MCAO (Figures 1A,B). Consistent with previous studies (Chang et al., 2011; Dziennis et al., 2011), we found that the expression of CD11b, which is a marker of M1-type microglial cells, was elevated in the peri-infarct region of the MCAO groups compared with that in sham group. Interestingly, we found that PDE1-B expression was also increased in the CD11b-positive cells (Figures 1C,D). These results suggest that ischemic brain tissue promotes PDE1-B expression in the M1 microglial cells.

### Vinpocetine Inhibits the OGD-Induced M1-BV2 Activation and Promotes M2 Phenotype

**FIGURE 2:**
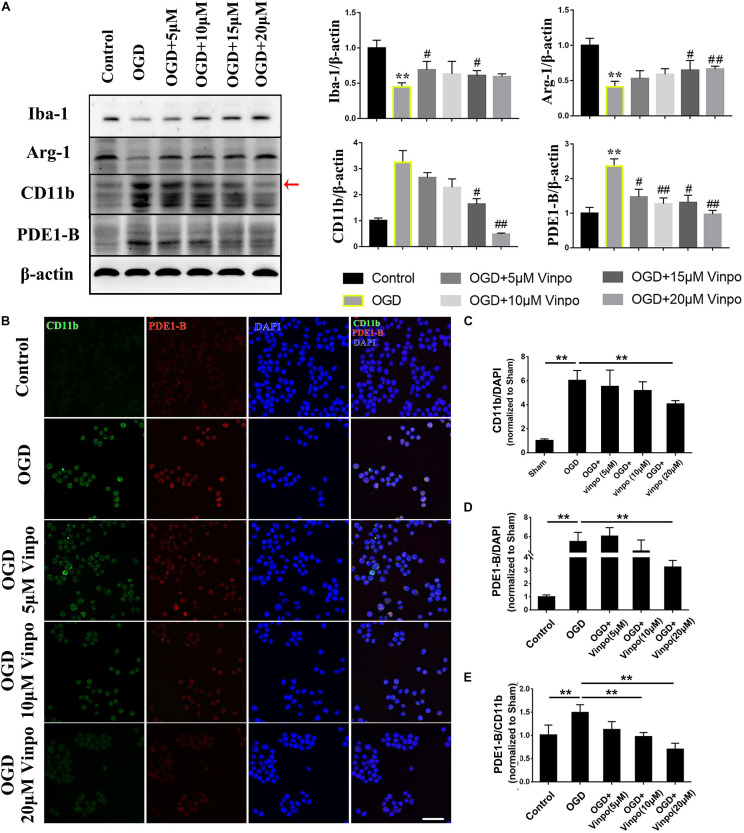
Vinpocetine treatment inhibits BV2 M1 and promotes M2 phenotype in OGD condition. **(A)** Representative immunoblots and quantification of Iba-1, Arg1, CD11b, PDE1-B in BV2 cells pre-treated with the indicated concentrations of vinpocetine for 24 h before OGD incubation for 3 h or in normal medium (control). Whole cell lysates were used for immunoblotting with antibodies against Iba-1, Arg-1, CD11b, PDE1-B, and β-actin. Each band has three repeated experiments for statistical analysis. *p < 0.05 and **p < 0.01 versus Control group; ^#^*p* < 0.05 and ^##^*p* < 0.01 versus OGD group. **(B)** Representative immunofluorescence images of CD11b and PDE-1B positive BV2 cells as treated in **(A)**. **(C–E)** Quantification of CD11b **(C)**, PDE1-B positive cells **(D),** and PDE1-B positive cells per CD11b positive cells **(E)**. **p < 0.01. Scale bar = 100 μm.

To investigate the role of PDE1-B in ischemic microglial cells, we employed an oxygen and glucose deprivation (OGD) model and used vinpocetine to selectively inhibit PDE1 (Hagiwara et al., 1984) in microglial BV2 cells. Consistent with our *in vivo* observation in Figure 1C, PDE1-B expression was increased in OGD-treated BV2 cells (Figure 2A). In addition, we examined the effect of vinpocetine on the polarity of BV2 cells under ischemic conditions using Iba-1, CD11b, and Arg-1 expression as the markers for general, M1 and M2 microglia, respectively. As expected, Iba-1 and Arg-1 were significantly decreased in the OGD-treated cells, while CD11b expression was elevated significantly (Figure 2A). Vinpocetine treatment significantly decreased PDE1-B protein expression, which was accompanied by repression of CD11b in a dose-dependent manner and enhancement of Arg-1 expression (Figure 2A). And the 20 μM vinpocetine treatment obtained a significant change when compared with OGD modeling cells without drugs. We further confirmed these observations with immunofluorescence analysis. As shown in Supplementary Figure S1, vinpocetine dose-dependently reversed the abundance of CD11b-positive cells and Arg-1-positive cells in OGD conditions. Co-staining analysis revealed that PDE1-B expression in CD11b-positive BV2 cells was reduced in a vinpocetine dose-dependent manner (Figures 2B–E). Our result in Figure 1 and others have shown that Iba-1 expression is increased in the activated microglia. In contrast, we found that in BV2 cells under OGD treatment, Iba-1 expression was decreased (Figure 2A, lane 2). This discrepancy may be due the different nature of the *in vivo* and *in vitro* experiments. It is conceivable that although BV2 cells were activated in OGD condition, the OGD-induced damage reduced the Iba-1 expression as reported previously (Lu et al., 2019; Xu et al., 2019). These results suggest that inhibition of PDE1-B by vinpocetine inhibit M1 microglia polarization in ischemic conditions.

### Vinpocetine Suppresses M1 BV2 Activation by Enhancing Autophagic Flux in Ischemic Condition

**FIGURE 3:**
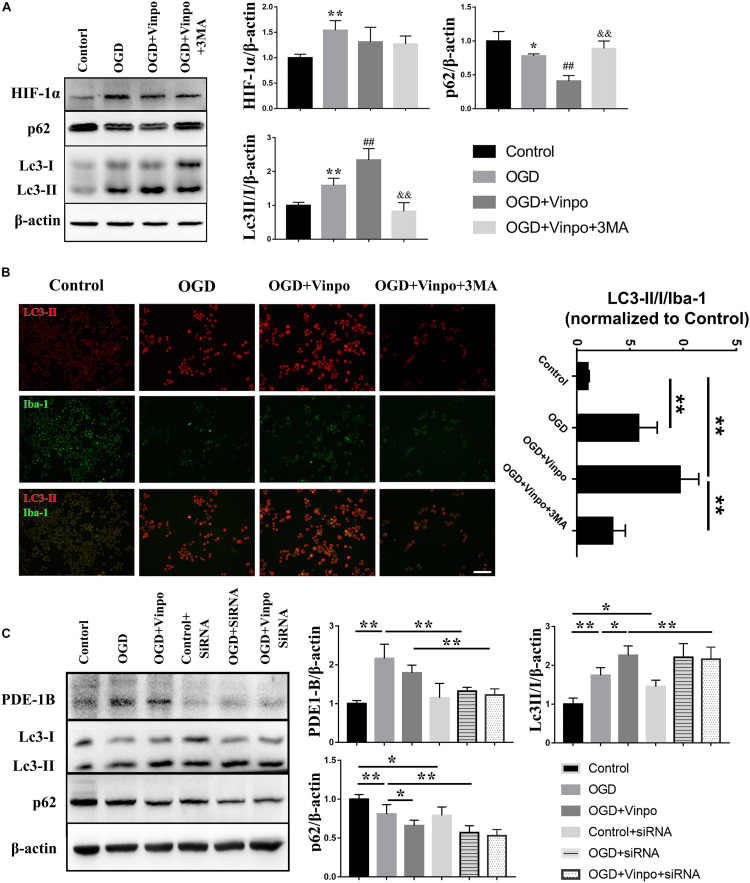
Vinpocetine treatment enhances autophagy in OGD-conditioned BV2 cells. **(A)** Representative immunoblots and quantification of HIF-1α, p62, LC3-I, LC3-II, β-actin in BV2 cells treated with vinpocetine (Vinpo) or/and 3-MA for 24 h after OGD incubation for 3 h or in normal medium (control). Whole cell lysates were used for immunoblotting. *p < 0.05 and **p < 0.01 versus control; ^#^*p* < 0.05 and ^##^*p* < 0.01 versus OGD; ^&&^*p* < 0.01 versus OGD + vinpo. **(B)** Representative immunofluorescence images of LC3-II and Iba-1 co-staining in BV2 cells as treated in **(A)**, and the quantification of the ratio of LC3-II positive BV2 cells to Iba-1 positive cells. **p < 0.01, Scale bar = 100 μm. **(C)** Representative immunoblots and quantification of cleaved caspase 3, Bax, Bcl-2 and β-actin in BV2 cells which pre-treated PED1-B siRNA or treated with vinpocetine (Vinpo) after OGD incubation or in normal medium (control). Whole cell lysates were used for immunoblotting. *p < 0.05 and **p < 0.01.

Oxygen-glucose deprivation (OGD) is known to strongly induce autophagy which is generally regarded as an adaptive mechanism for protection of cells from transient metabolic stress (Kroemer et al., 2010). We reasoned that the observed vinpocetine-enhanced-microglia viability under OGD condition may be resulted from the activation of autophagy. To test this hypothesis, we detected various autophagy marker proteins by western blot. As shown in Figure 3A, HIF-1α expression was highly increased in OGD treated BV2 cells, indicating the success of our OGD model. After OGD treatment, expression of p62 was declined and LC3-II/I ratio was significantly increased, demonstrating that the autophagic flux was elevated after OGD. We found that vinpocetine treatment further enhanced the LC3II/I ratio and decreased the p62 expression in BV2 cells. Moreover, blocking autophagy by inhibitor 3-Methyladenine (3-MA) led to abolishment of the vinpocetine-induced autophagy enhancement (Figure 3A). We further performed immunofluorescence staining and showed that LC3-II level was increased in Iba-1-positive cells after OGD treatment (Figure 3B). After vinpocetine treatment, the expression of LC3-II in Iba-1-positive cells was further increased compared with that in the OGD group. In agreement with the western blot results, 3-MA treatment completely eliminated the enhancement of autophagy by vinpocetine (Figure 3B). We further knocked down PDE1-B expression in BV2 cells by siRNA and found that the autophagy level of BV2 in both control and OGD groups were also increased to significantly (Figure 3C). These results imply that PDE1-B plays a critical role in negatively regulating autophagic activity in BV2 cells under ischemic conditions.

**FIGURE 4:**
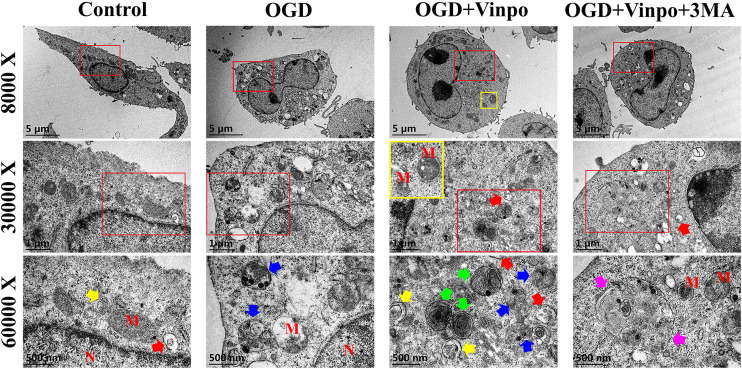
Transmission electron microscope images of the ultrastructure in different BV2 cells. Four groups BV2 cells has been shown (*N* = 3 cells per group), which incubated in normal medium (control), OGD condition for 3 h in the absence or presence of pretreatment with vinpocetine (Vinpo) or 3-methyladenine (3-MA) for 24 h. ‘M’ for mitochondria; ‘N’ for nucleus; blue arrows for autolysosomes; yellow arrows for early autophagosomes; green arrows for late autolysosomes; pink arrows for oversize autophagosomes; red arrows for endosomes.

To examine the changes of autophagic flux by vinpocetine treatment in more details, we used electron microscopy to investigate the effect of vinpocetine on autophagosome formation in BV2 cells (Figure 4). We observed that under normal condition, the cytoplasm of microglial cells appeared normal, with intact mitochondria and endoplasmic reticulum. After OGD treatment, however, a large-number of vacuoles appeared in the cytoplasm and the mitochondria became dissolved or fragmented. A large number of late autophagosomes (lysosome-containing autophagosomes, blue arrows) in the OGD BV2 cells were also observed. Notably, BV2 cells treated with vinpocetine exhibited more autophagosomes and endosomes (red arrows). Moreover, most mitochondria remained intact, and the number of autophagosomes in the cells increased prominently with the overall improved cell morphology, although mitochondrial dissolution was still observed. Under higher magnification, the early autophagosomes (microbubbles with intact membrane structures encapsulating certain contents without lysosomes, marked by yellow arrows), late autophagosomes (autolysosomes), and end-stage autophagosomes (microbubbles with intact membrane structures containing nested multilayer membrane structure, with or without lysosomes, marked by green arrows) were more prominently observed in the cells treated with vinpocetine. To determine whether the observed effect of vinpocetine was dependent on autophagic pathway, we used 3-MA to inhibit autophagy formation and mature in vinpocetine-treated cells and found that both of the number and size of autophagosomes were apparently reduced by the treatment of 3-MA. Thus, these results demonstrate that vinpocetine enhances autophagy in BV2 cells under OGD condition.

We further asked whether autophagy in BV2 cells was involved in mediating microglia polarization in ischemic conditions. We used Arg-1 and CD11b to determine the M2 and M1 microglial BV2 cells, respectively, and 3-MA to inhibit autophagy. Consistent with the results in Figure 2A, OGD significantly increased the expression of CD11b and decreased Arg-1 and Iba-1 expressions, and vinpocetine treatment blocked this change induced by OGD (Supplementary Figure S2A). Treatment of 3-MA significantly inhibited the effect of vinpocetine on suppressing microglia M1 polarization without the change of PDE-1B level (Supplementary Figure S2A). We performed immunofluorescence analysis and observed a similar effect of vinpocetine on BV2 polarization (Supplementary Figures S2B,C). These results demonstrate that vinpocetine suppresses M1 and promoted M2 microglia phenotype through, at least in part, positive regulation of autophagy.

### Vinpocetine-Treated BV2 Cells Alleviate OGD-Induced Neuronal Damage in Co-culture System

**FIGURE 5:**
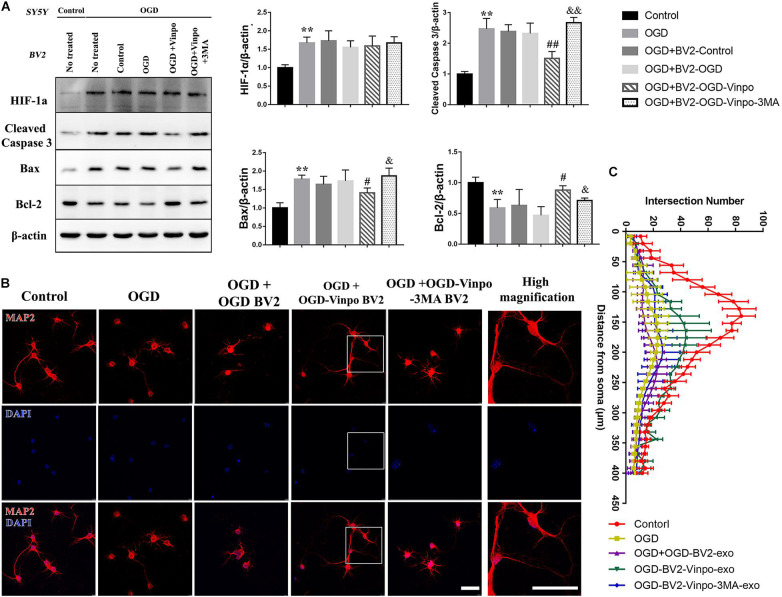
Vinpocetine-treated OGD-conditioned BV2 cells alleviates OGD-induced SH-SY5Y apoptosis and loss of neuron synapse. **(A)** Representative immunoblots and quantification of HIF-1α, cleaved caspase 3, Bax, Bcl-2, and β-actin in SH-SY5Y cells which were co-cultured with BV2 cells treated as described in Figure 3A. **p < 0.01 versus control; ^#^*p* < 0.05 and ^##^*p* < 0.01 versus OGD + OGD-BV2; ^&^*p* < 0.05 and ^&&^*p* < 0.01 versus OGD + OGD-vinpo-BV2. **(B)** Representative immunofluorescence images of MAP2 staining of primary mouse neurons co-cultured with the BV2 cells treated as described in **(A)**. **(C)** Quantification of intersection number of neurite in **(B)**. Scale bar = 100 μm.

Microglia play a crucial role in mediating neuronal survival after ischemic stroke. To examine the effect of vinpocetine on autophagy of microglia under ischemic conditions and the resulting influence on neuronal cell viability, we performed an *in vitro* Transwell assay using co-culture of BV2 cells and SH-SY5Y cells. BV2 cells were firstly incubated in OGD medium, or OGD in the presence of vinpocetine, or OGD in the presence of vinpocetine and 3-MA. Then these conditioned BV2 cells were co-cultured with SH-SY5Y cells in normal or OGD conditions. As expected, the expression of HIF-1α was increased in SH-SY5Y cells after OGD incubation (Figure 5A). The apoptosis level, as judged by cleaved caspase 3 expression, in SH-SY5Y cells was also elevated by OGD treatment. In OGD condition, SH-SY5Y cells that were co-cultured with control BV2 cells (BV2 conditioned in normal medium) or OGD-conditioned BV2 cells showed no significant change in cleaved caspase 3, Bax and Bcl-2 levels compared with no-BV2 control (Figure 5A lane 3&4 vs. lane 2). However, vinpocetine plus OGD-conditioned BV2 cells significantly alleviated SH-SY5Y apoptosis in OGD condition (Figure 5A, lane 5 vs. lane 4), as judged by cleaved caspase 3, Bax and Bcl-2 levels. We found that 3-MA treatment significantly reversed the effect of vinpocetine-treated OGD BV2 cells on SH-SY5Y cell viability. In addition, we performed TUNEL staining and confirmed the effect of vinpocetine on protecting SH-SY5Y cells in an autophagy-dependent manner (Supplementary Figure S3). These results imply that vinpocetine may regulate the secretion of certain cell permeable substances from the conditioned BV2 cells that influenced SH-SY5Y viability in OGD condition. We also examined the neurite morphology in the co-cultured primary mouse neuronal cells with the conditioned BV2 cells. Microtubule-associated protein 2 (MAP2) functions as a neuron-specific cytoskeletal protein marker for monitoring the dendritic structures. As shown in Figures 5B,C, MAP2 staining indicated that the morphology of neurons was significantly impaired in OGD condition. Co-cultured with OGD-conditioned BV2 cells further reduced the number of the dendrites. However, treatment of neuronal cells with vinpocetine plus OGD-conditioned BV2 cells significantly alleviated the change of dendritic structural impairment in neurons, and 3-MA reversed the effect of vinpocetine. These results suggest that the secretion from OGD-conditioned BV2 cells may further deteriorate the neurite morphology, while vinpocetine treatment in the OGD-conditioned BV2 cells mitigates the deformation of neurites in a BV2 autophagy-dependent manner.

### Vinpocetine Treatment Regulates OGD-Conditioned BV2 Cell-Derived Exosomes Which Influences Neuronal Viability and Neurite Morphology

**FIGURE 6:**
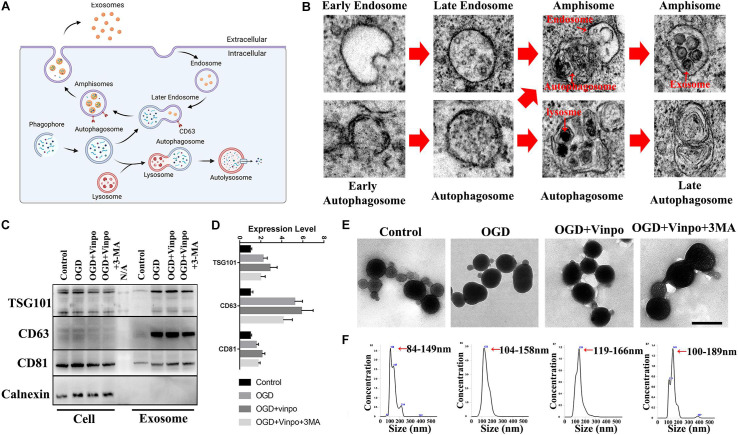
Identification of the exosomes derived from OGD-conditioned BV2 cells. **(A)** Illustration for the relationship between exosome biogenesis and autophagic flux. **(B)** Scanning electron microscopic images showing the existence of different states of autophagosome formation and exosome packaging in amphisome in BV2 cells incubated in OGD for 3 h. **(C)** Representative immunoblots and quantification of TSC101, CD63, CD81, and Calnexin in whole cell extract (Cell) and the purified exosomes from BV2 cells which were treated as described in Figure 3B. **(D)** Quantification of TSG101, CD63, CD81 in **(C)**. **(E)** Transmission electron microscopic images of the purified exosomes. **(F)** Nanoparticle tracking analysis of the purified exosomal size (*N* = 3 repeated detections per group). Scale bar = 200 nm.

The above results implied that certain secretory substance from the conditioned BV2 cells played a role in regulation of recipient cell viability in OGD condition. We hypothesized that exosomes may be the candidate because accumulating evidence has revealed the functions of exosomes in microglia-neuron communication (Men et al., 2019). Exosomes are produced by inward budding into the early endosome, resulting in maturation into multivesicular bodies (MVB)/late endosome. After their formation, the late endosome can either fuse with the lysosome to degrade its content or fuse with the plasma membrane to release the intraluminal vesicles (ILVs) as exosomes (Song L. et al., 2019). It has been proposed that exosome biogenesis and secretion were closely associated with autophagic flux (Gudbergsson and Johnsen, 2019; Jeppesen et al., 2019) (Figure 6A). To determine whether these phenomena occurred in our *in vitro* system, we examined the subcellular structures of BV2 cells cultured in OGD condition by EM analysis. Indeed, we observed various intracellular vesicles which were related to autophagic flux and exosome biogenesis (Figure 6B). Thus, these results confirmed the existence of exosome formation in the BV2 microglial cells incubated in OGD condition. Our observations further suggest that autophagosomes could either fuse with lysosome or late endosomes to form autophagosomes or amphisomes, respectively, since amphisomes could fuse with plasma membrane of microglia to release exosomes, while autophagosomes could proceed to autolysosome formation in OGD condition.

Next, we tried to characterize the exosomes released from the BV2 cells under ischemic condition. We extracted and purified exosomes that were secreted by different conditioned BV2 cells. Results of the nanoparticle tracking analysis (NTA) showed that the size of isolated microvesicles ranged from 84 to 189 nm (Figure 6F) which were slightly bigger than the usual exosomal size of 40–160 nm (Jeppesen et al., 2019; Kalluri and LeBleu, 2020). This was likely due to the method we used to purify exosomes. To enhance the yield of exosomes, we used the precipitation-based polyethylene glycol (PEG) method which is known to slightly enlarge the size of exosomes for their precipitation fur the isolation process (Liu et al., 2017). In addition, the TEM results showed that the microvesicles we isolated were generally cup-shaped and round particles with a complete membrane structure (Figure 6E). These characteristics were consistent with the morphology and size of exosomes (Jeppesen et al., 2019). For further verification, we examined the exosome marker proteins in microvesicle lysates by western blot and found that TSG101, CD63, and CD81 were detected, but the endoplasmic reticulum-associated protein Calnexin was not detected in the extracts (Figure 6C). These results indicate the success of our exosome isolation from BV2 cells. Then, we compared the exosomes isolated from different conditioned BV2 cells. NTA results in Figure 6F showed that the average exosome size was increased in the OGD-conditioned BV2 cells. Vinpocetine treatment further increased the size of the released exosomes. Exosomes exhibited largest size after BV2 cells treated with 3-MA and vinpocetine in OGD medium (Figures 6E,F). In addition, TEM image of exosomes from the OGD + vinpo + 3MA treatment implied the possibility of fusion of exosomes (Figure 6E). By western blot analysis, we found that all of the expression of exosome marker proteins TSG101, CD63, and CD81 were increased after OGD treatment. Consistent with the NTA results, vinpocetine treatment further increased the exosome specific proteins slightly. However, those exosome specific protein levels were reduced by 3-MA treatment (Figures 6C,D). These results suggest that vinpocetine-enhanced autophagy in microglia affects the physical property and loaded contents of exosomes.

**FIGURE 7:**
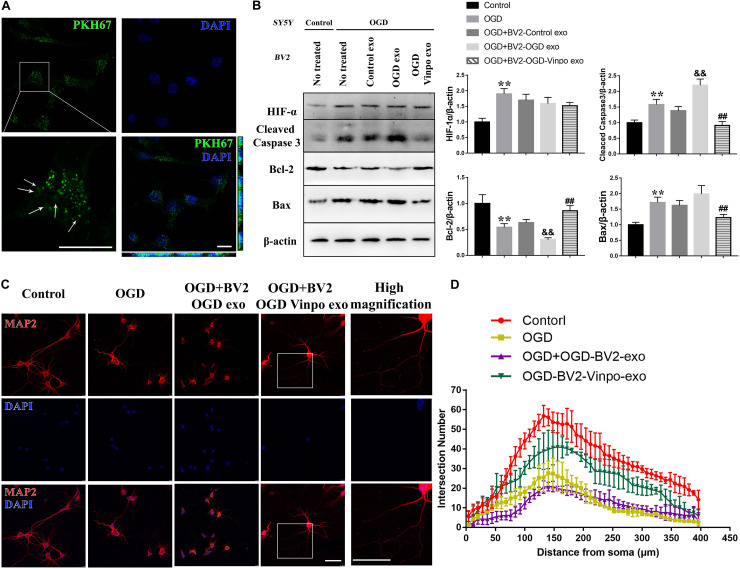
The exosomes derived from vinpocetine-treated OGD-conditioned BV2 cells ameliorates OGD-induced SH-SY5Y apoptosis and loss of neuron synapse. **(A)** Exosomes purified from BV2 cells cultured in normal medium were labeled with PKH67 before incubated with SH-SY5Y cells. The arrows indicate the labeled exosomes were taken up inside the SH-SY5Y cells. **(B)** Representative immunoblots and quantification of HIF-1, cleaved caspase 3, Bcl-2, Bax and β-actin in SH-SY5Y cells which were incubated with the purified exosomes isolated from different conditioned BV2 cells treated with vinpocetine (Vinpo) or not for 24 h after OGD or normal incubation (control). **p < 0.01 versus control; ^&&^*p* < 0.01 versus OGD; #*p* < 0.05 and ##*p* < 0.01 versus OGD + BV2-OGD-exo; ^&^*p* < 0.05 and ^&&^*p* < 0.01 versus OGD + BV2-OGD-vinpo exo. **(C)** Representative immunofluorescence images of MAP2 staining of primary mouse neurons incubated with the purified exosomes isolated from the BV2 cells treated as described in **(B)**. **(D)** Quantification of intersection number of neurite in **(C)**. Scale bar = 100 μm.

To determine whether the conditioned BV2-derived exosomes *per se* were sufficient to cause the observed effects, we directly added the purified exosomes isolated from different conditioned BV2 cells to SH-SY5Y cells and primary neuron, and examined the SH-SY5Y cell survival and neurite morphology. We first determined whether the purified exosomes could be taken up by the recipient neuronal cells. To this end, the isolated exosomes were firstly stained with PKH67, washed and then added into the neuronal cell culture in order to distinguish the exogenous exosomes from the endogenous ones in neurons. As shown in Figure 7A, the distribution of pixel color dots along the *x*- and *y*-axes of that images indicate that the labeled exosomes were significantly enriched inside the recipient neurons.

We then treated the neuronal SH-SY5Y cells with the isolated exosomes from different conditioned BV2 cells and determined SH-SY5Y cell viability. As shown in Figure 7B, although the control-BV2- or OGD-conditioned BV2-derived exosomes did not significantly change the maker protein expression levels of apoptosis in SH-SY5Y cells in OGD condition, exosomes purified from the vinpocetine-treated OGD-conditioned BV2 cells significantly decreased apoptosis in SH-SY5Y cells in OGD condition as judged by the reduced expression of cleaved caspase-3 and Bax, and enhanced expression of Bcl-2 (Figure 7B). Consistently, the cell damage detection, TUNEL staining also indicated a similar effect on cell damage (Supplementary Figure S4). We also detected the effect of BV2-derived exosomes on neurite morphology. Immunofluorescence results showed that the treatment of isolated exosomes purified from OGD-conditioned BV2 cells caused a further impairment of neurite structure. The exosomes generated from vinpocetine-treated OGD-conditioned BV2 cells significantly alleviated the structural impairment of neurites (Figures 7C,D). These results suggest that vinpocetine regulates the neuroprotective property of OGD-conditioned BV2-derived exosomes which influenced SH-SY5Y survival and neurite structure in OGD condition.

### Brain Injection of Exosomes Is Sufficient to Affect Neuronal Survival

**FIGURE 8:**
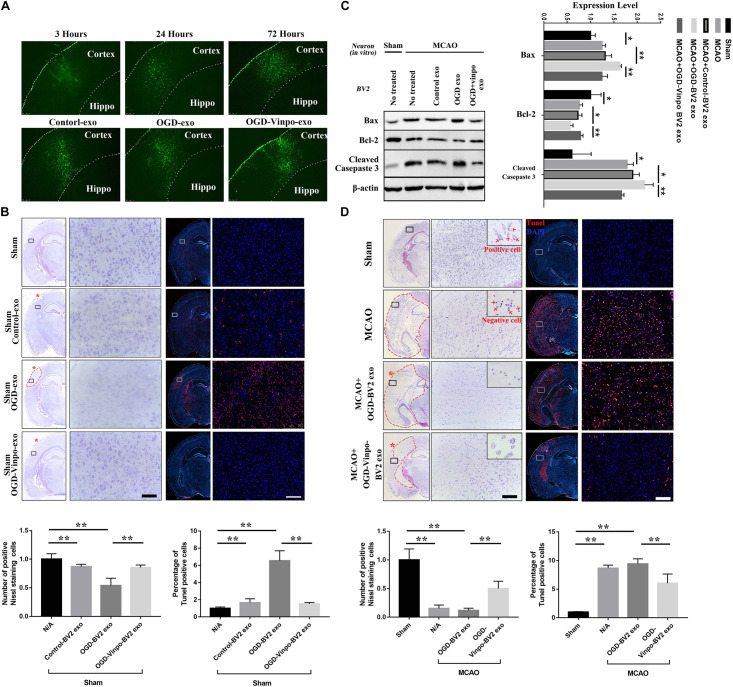
Effect of exosomes derived from different condition BV2 on brain tissue.**(A)** Distribution of exosomes derived from microglia in normal condition (Control-exo) 3, 24, and 72 h after injection to the cortex (upper panels). Distribution of exosomes isolated from different conditioned BV2 at 72 h after cortical injection (bottom panels). **(B,D)** Nissl and TUNEL staining of brain sections from normal or transient MCAO mice (*N* = 3) injected with exosomes purified from different conditioned BV2 for 72 h. **(B)** For normal mice, **(D)** for transient MCAO mice. The red asterisks indicate the cortical injection sites. **(C)** Representative immunoblots and quantification of Bax, Cleaved Casepase 3, Bcl-2 and β-actin expression levels in normal or ischemic cortical tissue extracts from mice injected with the purified exosomes isolated from BV2 cells as described in Figure 7B for 72 h. *p < 0.05 and **p < 0.01.

To verify the exosome function *in vivo*, we isolated exosomes from the various conditioned BV2 cells, and injected those exosomes directly into the left cortex of normal adult mice. We then monitored the localization of exosomes after injection in the cortex at different time points. The immunofluorescence imaging results showed that the PKH67-labeled exosomes were accumulated in the cortex over times, and peaked at after 72 h of injection (Figure 8A, upper panel). The distribution of the injected exosomes collected from different conditioned BV-2 cells in the cortex of normal mice after 72 h showed no observable difference (Figure 8A, lower panel). Nevertheless, the Nissl and TUENL staining results in Figure 8A indicated that the OGD-conditioned BV2-derived exosomes (OGD-exo) significantly reduced the neuronal cell number and increased TUNEL-positive cells around the injected area in normal mice brain, further supporting the notion that the OGD-conditioned BV2-derived exosomes causes neuronal loss. In contrast, injection of the exosomes derived from vinpocetine-OGD-conditioned BV2 reversed OGD-BV2 exosomes induced neuronal damage (Figure 8B). To examine whether the vinpocetine-OGD-BV2 exosome had a protective effect against stroke-induced neuronal damage, we injected the OGD-BV2 exosomes and the vinpocetine-OGD-BV2 exosomes to the cerebral cortex of mice subjected to transient MCAO. After 72 h of MCAO, the cortical tissues were collected for western analysis of apoptosis. We confirmed that MCAO caused significant increased apoptosis in the brain tissue (Figure 8C). The exosomes derived from OGD-conditioned BV2 further enhanced the protein levels of apoptotic markers, while exosomes isolated from the vinpocetine treated OGD condition BV2 cells significantly reduced apoptosis (Figure 8C). In addition, we used Nissl and TUNEL stainings to confirm the effect of vinpocetine on rescuing the neuronal loss and reducing cell damage in the cortical tissue induced by MCAO (Figure 8D). These results indicate that the exosome derived from OGD-BV2 causes neuronal damage whereas exosomes derived from vinpocetine-treated BV2 protects the neurons against stroke-induced damage.

## Discussion

Phosphodiesterase enzyme is a phosphodiesterase that is also known as calcium- and calmodulin-dependent phosphodiesterase. By catalyzing the hydrolysis of cAMP and cGMP, PDEs limit intracellular levels of cyclic nucleotides and thus regulate the amplitude, duration, and compartmentation of cyclic nucleotide signaling. They participate in many cell processes including platelet functions^1^, stabilization of the blood-brain barrier^2,3^, and inflammation^4^. The superfamily of PDE enzymes is classified into 11 groups, including PDE1-PDE11 in mammals^5^, and PDE1 is currently the best characterized one^6^. There are three PDE1 isoforms have been identified, and PDE1-B is mainly distributed in cerebral cortex^7^ Vinpocetine is a well-known PDE1 inhibitor that is a synthetic derivative of the vinca alkaloid vincamine. Previous studies have reported the protective effects of vinpocetine on neurons and other brain cells after ischemic injury in rodents (Sauer et al., 1988) and humans (Szilagyi et al., 2005; Vas and Gulyas, 2005; Zhang W. et al., 2016). It can serve as a neuroprotective drug that possesses anti-inflammatory effects against cognitive impairment (Gulyas et al., 2011; Ali et al., 2019a, b). Recent studies have reported that vinpocetine inhibits TLR4 expression in microglial cells after OGD, leading to a decreased inflammatory response by downregulation of TLR4/MyD88/NF-κB pathway after cerebral ischemia-reperfusion injury (Wu et al., 2017). Interestingly, this vinpocetine effect is independent of its well-known inhibitory effects on phosphodiesterases (Jeon et al., 2010). Nevertheless, whether and how PDE1B mediates neuroprotective mechanism in stroke remains incompletely understood.

**FIGURE 9:**
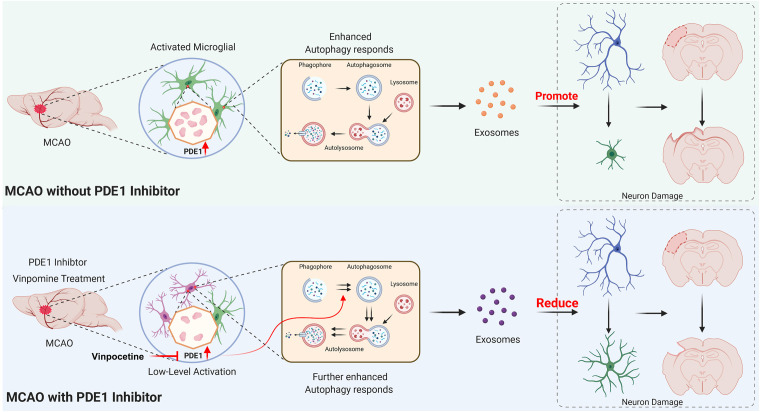
Working model of the role of PDE1-B and the mechanism of vinpocetine in protection against neuronal damage. See main text for detailed description.

Several lines of evidence have indicated that the PDE1 inhibitor vinpocetine suppresses the release of inflammatory cytokine and chemokines from multiple cell types by targeting NF-κB-dependent inflammation pathway (Liu et al., 2017; Zhang F. et al., 2018) and involving regulation of neuronal plasticity in acute ischemic stroke patients (Medina et al., 2006). In this study, we further demonstrate a novel mechanism by which vinpocetine regulates microglia-neuron communication through alteration of autophagy in microglial cells. As proposed in our working model in Figure 9. We demonstrated that PDE1-B expression is significantly increased in the activated M1 microglia in the ischemic brain tissue. To investigate the potential role of PDE1-B and the effect of its inhibitor vinpocetine on microglial function, we used BV2 cells and the OGD model to examine the microglial response under ischemic stroke condition *in vitro*. Vinpocetine inhibits BV2 microglia M1 and promotes M2 phenotype in ischemic OGD condition. In addition, our observation suggested that inhibition or knockdown of PDE1-B enhances autophagy in OGD-conditioned BV2 cells, which is associated with the release of exosomes that protects neurons against OGD-induced damage. Finally, we directly showed in animal stroke model that the exosomes released from the microglia BV2 cells in OGD condition causes neuronal damage while exosomes released from the vinpocetine-treated BV2 cells promotes neuroprotection against ischemia-induced damage. These results reveal a novel mechanism of microglia-neuron communication through microglial exosomes. Inhibition of PDE1B alters microglial autophagic flux for microglial exosome synthesis and release which plays critical role in neuronal survival and neurite structure. Although it is still unknown how the change of autophagic flux could modulate the exosome contents and release from microglia, the exosomes released from microglia under the ischemic condition enter neurons and exert their neuroprotective effect against ischemia-induced damage.

As known, OGD is a known inducer of autophagy (Zhou et al., 2017; Perez-Alvarez et al., 2018). It is an active defense response in cells that protects cells from damage to a certain extent (Fu et al., 2016). In the early stage of cell damage, autophagy promotes cell repair and survival and inhibits apoptosis (Kroemer et al., 2010). However, with further exacerbation of cell damage, the incrementally enhanced autophagy level of the cell will result in excessive self-digestion, and further lead to the cell death finally, which is called autophagy-dependent cell death (Li et al., 2015; Song S. et al., 2017; Cristofani et al., 2018). This is the dual role of autophagy in cell survival. Interestingly, we observed an uncommon regulation of vinpocetine in OGD- BV2 cells. We showed that vinpocetine treatment further enhances autophagic flux. This effect of vinpocetine is resulted from the inhibition of PDE-1 as knockdown of PDE-1 produces a resemble effect on alteration of autophagy.

According to previous studies (Dower et al., 2018; Minakaki et al., 2018; Jeppesen et al., 2019), autophagosomes have two different fates. The first is fusing with lysosomes to form autolysosomes, which degrade and recover contents. And the second is combining with later endosomes and fuse with the cell membrane, further releasing contents from the cells via extracellular vesicle. However, these two processes are not independent, they share some common mechanisms and affect each other in some cases. For example, the H + ATPase inhibitor bafilomycin A1 (BafA1) prevents the fusion of autophagosomes and lysosomes and further promotes the secretion of cytosolic pro-IL-1β to regulate the function of recipient cells (Klionsky et al., 2016). Inhibiting the autophagy-lysosome pathway alters extracellular vesicle biochemical profiles and enhances synuclein alpha release via exosomes by increasing extracellular shuttling of multivesicular body contents and further promotes synuclein alpha cell-to-cell transfer (Minakaki et al., 2018). Therefore, autophagy has a close relationship with exocytosis and may be involved in the regulation of signal transmission between cells.

Exosomes are one of the extracellular microvesicles with a diameter of approximately 40–160 nm (Jeppesen et al., 2019; Kalluri and LeBleu, 2020), Exosomes are released from cells, contain large amounts of proteins, nucleic acids, and are further taken up by targeted cells to release signal regulators and induce powerful biological downstream effects (Mathieu et al., 2019; Pluchino and Smith, 2019). As previously mentioned, exosomes generation and autophagosomes are involved in similar mechanisms (Minakaki et al., 2018; Jeppesen et al., 2019). The endosomes formed by the invagination of the cell membrane and service as the precursors of exosomes, they carry specific molecular markers, including CD63, which is also the specific membrane protein of exosomes. Late endosomes can fuse with autophagosomes and form amphisomes that have both the exosome marker CD63 and the autophagosome marker LC3. Amphisomes further release the exosomes to the extracellular space. Thus, exosomes can transport the cargo of autophagosomes to the recipient cells by forming amphisomes, and autophagy may also serve as a critical factor in regulating exosome generation. In the presented study, we isolated the exosomes secreted by BV2 cells in different conditions, and found that OGD and vinpocetine treatments altered the autophagic flux and the size of exosomes released from BV2 cells. Consistently, it has been reported that environment conditions could alter the physical and chemical properties of exosomes (Hajrasouliha et al., 2013). It should be noted that the exosomes we isolated from BV2 cells were slightly larger than the usual size of exosomes because we used the precipitation-based polyethylene glycol method for maximizing the yield in our experiment. Unexpectedly, inhibition of autophagy by 3-MA further increases the exosome volume. We noticed that the size of exosomes could be ranged from 100 to 189 nm. We speculate that 3-MA may inhibit the formation of autophagosomes (Seglen and Gordon, 1982), resulting in the observed oversized autophagosomes (Figures 6E,F) which may imply the fusion of autophagosomes and endosomes. This will require further characterization in the future research to clarify the significance and the identify of loaded materials in these particles. To gain more insight into the underlying mechanisms, we are conducting RNA-sequencing and proteomics analysis in our laboratory in microglial exosomes under different vinpocetine treatment conditions.

It should be noted that we only used male mice in this study in order to avoid the effect of sex hormone (Spychala et al., 2018; Zhang H. et al., 2018; Otxoa-de-Amezaga et al., 2019; Xu et al., 2019; Al Mamun et al., 2020; Ye et al., 2020). It has been reported that estrogen receptors (ERs) are widely distributed in the brain, present on both neurons and glia, and expressed by both sexes (Rettberg et al., 2014; Hewitt and Korach, 2018). The sex hormone is involved in the regulation of glucose transport, aerobic glycolysis, and mitochondrial function and ATP synthesis (Liu et al., 2014). Therefore, it is likely that the periodic changes of hormone levels in female mice may affect the neuronal damage and recovery after stroke. While we showed the role of microglial exosome and the mechanisms concerning the effect of vinpocetine in male mice, the sex discrepancy and the effect of vinpocetine on female mice are needed to be further investigated in the future.

In summary, we discover a novel neuroprotective mechanism of vinpocetine. By inhibiting PDE1-B in microglial cells, vinpocetine does not only inhibit the M1 microglial phenotype but also enhance autophagic flux which is associated with the alteration of exosomal contents and properties for protecting the survival and neurite structure of neurons against ischemic stroke. However, there are outstanding questions remain unanswered. For example, what are the identity of loading materials inside the microglia-derived exosomes? What alterations, if any, of these materials after vinpocetine treatment? A multiple dimensional omics analysis such as RNA-sequencing, proteomics and lipidomics will be required to more comprehensively analyze the exosome contents and their changes that are related to their neuroprotective functions. Answers to these questions would be important for uncovering the exact pathophysiological roles of microglial exosomes in ischemic stroke and the development of effective therapeutic drugs for treatment of this devastating disease.

## Materials and Methods

### Animals

Adult 6- to 8-week-old C57/B6 male mice (20–24 g, *N* = 12 in each group) were purchased from Guangzhou University of Chinese Medicine. The *in vivo* experiment was approved by the local medical ethics committee. All animal procedures were performed in strict accordance with the Guide for the Care and Use of Laboratory Animal by International Committees and Reporting of *In Vivo* Experiments (ARRIVE) guidelines and under the supervision of the Experimental Animal Ethics Committee of Jinan University (Project ID: 0201028-03). Endeavors were made to reduce the total number of animals used as well as their potential pain and suffering. All animal experiments involved in this study have been approved by the Experimental Animal Ethics Committee of Jinan University. Male mice were used in this study considering that estrogen receptors (ERs) are widely expressed in the neurons and glial cells in the brain (Rettberg et al., 2014; Hewitt and Korach, 2018). ER activation is known to regulate glucose transport, aerobic glycolysis, and mitochondrial functions (Rettberg et al., 2014). The periodic changes of hormone levels in female mice may affect the stroke-induced damage and recovery.

### Middle Cerebral Artery Occlusion Surgeries

For distal MCAO, the mice (*n* = 3 mice per group) were anesthetized with isoflurane gas (4% induction anesthesia, 1.5% maintenance anesthesia; RWD Life Sciences, Shenzhen, China). An incision between the left eye and ear was made and the temporal muscle was cut and separated to expose the left side of the skull. The middle cerebral artery (MCA) was identified through the semi-translucent skull, and a burr hole (2- to 3-mm diameter) was drilled to expose the M1 portion as large as possible. The MCA was occluded by electrocoagulation with blunted needle. Saline was applied to the surgical area throughout the procedure to prevent heat injury and dehydration. Finally, the temporal muscle and scalp were sutured and the wound was sterilized. Body temperature was maintained at 37°C after surgery. Control mice were conducted the same experiment except for the MCA occlusion.

For transient MCAO model, mice (*n* = 3 mice per group) were anesthetized with isoflurane gas. MACO model was established by using intraluminal suture. Briefly, Under the microscope, the left common carotid artery (CCA), external carotid artery (ECA), and internal carotid artery (ICA) were isolated in the neck, a silicon-coated nylon suture was carefully introduced into ECA, then went into the internal carotid artery to 11 mm from the carotid artery bifurcation or resistance exist for blocking the MCA at the beginning of the MCA. MCA was blocked for 40 min, and then remove the intraluminal suture from MCA. Finally, the temporal muscle and scalp were sutured and the wound was sterilized. Control mice were conducted the same experiment except for the MCA occlusion.

### Stereotactic Injection

Adult C57/B6 male mice (6- to 8-week-old, 22-24 g, *N* = 4) were randomly grouped and were anesthetized with isoflurane gas. The animals were placed in a stereotactic frame (RWD Life Sciences, Shenzhen, China). Exosomes (4 μl at 10 μg protein/μl) from different groups were stereotactically injected into the cortex of mice at the following two coordinates relative to bregma: the first site is anteroposterior (AP) 1.75 mm, mediolateral (ML) 3.0 mm, and dorsoventral (DV) 0.9 mm; the second site is AP 2.25, ML 3.0 and DV 1.0 mm. The frozen sections of the brain were obtained as described above. For the fluorescence slices of labeled exosomes, they are directly observed under the microscope without processing, and the other slices shall be processed accordingly.

### Cell Culture and Treatment

SH-SY5Y nerve cells and BV2 microglia (BV2) were purchased from ATCC. SH-SY5Y cells and BV2 cells were cultured in DMEM (Gibco, United States, Cat No. C11995) supplemented with 10% FBS and 1% penicillin-streptomycin in a 5% CO_2_ incubator at 37°C.

For primary neuron culture (Fath et al., 2009), the cortical neurons cells (biological triplicates, *n* = 3) were dissociated from the cerebral cortex of C57/B6 mice, and incubated in DMEM/F12 medium with 5% FBS after separation (Gibco, United States, Cat No. C11330500) for 4 h. Then changed the medium to neurobasal medium (Gibco, United States, Cat No. 21103049) with 2% B27 (Gibco, United States, Cat No. 177504044) and incubated for another 7 days at 37°C in a 5% CO2 incubator.

For siRNA transfection, BV2 cells were seeded in 6 cm culture plate (2 × 105 cells per well) in 4 mL antibiotic-free DMEM supplemented with 10% FBS. Once the cells reached 30–50% confluence, they were transfected siRNA using the riboFect^TM^ CP Transfection Kit (RiboBio, China) according to the manufacturer’s instructions. Forty-eight hours after transfection, the cells were used in further experiments. Small interfering RNA (siRNA) targeting PDE1 was purchased from RiboBio Company (Guangzhou, China), and the sequence have shown below:

PDE1-B siRNA: CTGGAGAATCACCACATCA

For OGD model, cells were maintained in an OGD environment, as described (Chen et al., 2016; Zang et al., 2017). Briefly, when the cell density reached 60–70%, the cells were washed twice in PBS (BI, Israel), glucose-free DMEM (Procell, China, Cat No. PM150270) was added, and the cells placed in a hypoxia chamber (95% N_2_, 5% CO_2_, and 0.1% O_2_). SH-SY5Y cells were incubated in OGD condition for 6 h, and BV2 cells were incubated for 3 h. BV2 cells in the vinpocetine group were pretreated with vinpocetine for 24 h before OGD incubation. At the end of OGD incubation, the cells were maintained in regular medium in a normal atmospheric air incubator and treatment with different drugs. Vinpocetine was obtained from MCE (United States, Cat No. HY-13295), 3-MA was purchased from Aladdin (China, Cat No. 5142-23-4). Biological triplicates were examined for each experiment.

### Transwell Co-culture System

Transwells were used to perform BV2-SH-SY5Y co-culture experiments. For transwell experiments, 1 × 10^5^ BV2 cells were seeded on 6-well inserts with 0.4 μm pores in normal culture medium, and 4 × 10^5^ SH-SY5Y cells were cultured in another 6-well plate for 24 h. After OGD incubation and treatment with different drugs, the inserts containing the BV2 cells were placed on the wells containing SH-SY5Y cell with or without OGD incubation for 24 h. Biological triplicates were examined for each experiment.

### Exosome Isolation and Labeling

Before exosome isolation, the cell medium was replaced with basic medium without FBS or exosomes and maintained for 40 h. Exosome isolation was performed as described in previous studies (Lobb et al., 2015; Zhang X. et al., 2019). After 48 h, the cell medium was collected and centrifuged. First, the medium was centrifuged at 200 *g* for 10 min to remove floating cells. Then, the medium was centrifuged at 2000 *g* for 20 min twice to remove cellular debris and was passed through a 0.22 μm filter (Millipore, United States). Next, the filtered medium was added to the 10000 MWCO centrifugal filter (Millipore, United States, Cat No. UFC901096) and centrifuged at 4000 × *g* for 35 min at 4°C. Finally, approximately 300 μl was enriched, and then the EXO Quick-TC Exosome Isolation Reagent (System Biosciences, United States, Cat No. EXOTC10A-1) was added to the concentrated solution. Following the reagent instructions, we harvested exosomes the next day. The exosomes were identified by electron microscopy, western blotting and NTA using a Nanosight NS300 (Malvern, United Kingdom). The results of each image and western bolt bands were independently repeated for three times.

To verify that SH-SY5Y cell or cortex tissues internalize exosomes, we stained exosomes with green fluorescence PKH67 (Sigma-Aldrich, United States, Cat No. mini67). We have made a few modifications based on the operation manual (Zhang X. et al., 2019). Briefly, exosome precipitates were suspended in 500 μl Diluent C, and then 500 μl Diluent C and 2 μl dye mixture were added. Subsequently, we mixed the sample by pipetting for 5 min at room temperature. Then, 10 ml complete medium which had passed through the ultrafiltration tube were added to stop the staining for 1 min. The exosomes were retained using ultrafiltration tubes and washed two more times in complete medium and once in PBS. Finally, the enriched liquid in the ultrafiltration tube was added to the cells (Figure 7A) or stereotactic injection into cortex (Figure 8A, *N* = 3). After cell fixation with 4% PFA and DAPI staining, the uptake of exosomes was observed under a laser transmission confocal microscope (Leica, United States).

### Transmission Electron Microscopy

Cells samples were fixed by immersion in 2.5% glutaraldehyde in 0.1 M Sorensen buffer, postfixed in 1% osmium tetroxide, and stained in 3% uranyl acetate. Then, the cells were dehydrated in ethanol and embedded in Epon. Ultra-thin sections were poststained with uranyl acetate and lead citrate and examined using a Philips CM100 electron microscope at 60 kV. Images were recorded digitally with a Kodak 1.6 Megaplus camera system that was operated using AMT software (Advanced Microscopy Techniques, Danvers, MA, United States).

For exosome samples, 10 μl fresh exosomes were dripped onto the carbon-coated copper grid for 10 min to dry and then fixed with 2.5% glutaraldehyde for 10 min. The copper grid was washed with a water drop and negatively stained with 1% phosphotungstic acid for 30 s. Images were recorded digitally with a Kodak 1.6 Megaplus camera system that was operated using AMT software. The results of each TEM image were independently repeated for three times.

### Immunofluorescence and Immunohistochemistry

The tissue samples were isolated after transcardial perfusion with 0.9% NaCl and fixation with 4% PFA. Then, the brains were cut into 10 μm frozen sections. For immunohistochemical staining, the sections were incubated in 0.3% H_2_O_2_ for 15 min to block endogenous peroxidase activity and permeabilized with PBS containing 1% Triton X-100 for 20 min. Then, the sections were blocked in 5% bovine serum albumin (BSA) for 30 min at room temperature. Without washing, the sections were incubated with the primary antibodies (Table 1) overnight at 4°C and incubated with biotin-conjugated secondary antibodies at 37°C for 30 min the next day. The immunoreaction was detected by horseradish peroxidase-conjugated antibody at 37°C for 30 min and visualized with diaminobenzidine (DAB). For immunofluorescence staining, sections were first incubated with a primary antibody mixture for 24 h at 4°C, followed by incubation with a mixture of fluorescent secondary antibodies for 1 h at room temperature. The images were acquired using an intelligent inverted fluorescence microscope (Leica, United States) and confocal laser microscopy (Zeiss LSM 510, Oberkochen, Germany). Three mice in each group were used for immunohistochemical and immunofluorescence assay (distal MCAO). Three independent fields were selected in each repeat experiment and the proportion of positive cells was counted for statistical analysis.

**TABLE 1:** Information of the primary antibodies used in this study.

**Antibody**	**Specificity**	**Type**	**Item numbers**	**Dilution**	**Source**
β-actin	β-actin	Monoclonal	#3700	1:10000 WB	Cell Signaling
Bcl-2	Total Bcl-2	Polyclonal	#15071S	1:1000 WB	Cell Signaling
BAX	BAX	Monoclonal	#5023S	1:1000 WB	Cell Signaling
Cleaved caspase 3	Cleaved caspase 3	Polyclonal	#9664	1:1000 WB	Cell Signaling
Iba-1	Iba-1	Monoclonal	#sc-32725	1:100 WB/1:10 IHC/1:10 IF	Santa Cruz
CD11b	CD11b	Polyclonal	#BMS104	1:1000 WB/1:100 IF	Invitrogen
Arg-1	Arginase-1	Polyclonal	#ABS535	1:1000 WB/1:100 IF	Sigma-Aldrich
PDE1-B	PDE1-B	Polyclonal	#ab14600	1:1000 WB/1:100 IF	Abcam
P62	SQSTM1/p62	Polyclonal	#23214	1:1000 WB	Cell Signaling
LC3I/II	LC3I/II	Polyclonal	#4108	1:1000 WB	Cell Signaling
LC3II	LC3II	Polyclonal	#3868S	1:100 IF	Cell Signaling
HIF-1α	HIF-1α	Monoclonal	#36169S	1:1000 WB	Cell Signaling
TSG101	TSG101	Polyclonal	#ab125011	1:1000 WB	Abcam
CD63	CD63	Polyclonal	#ab59479	1:1000 WB	Abcam
CD81	CD81	Monoclonal	#10037S	1:1000 WB	Cell Signaling
Calnexin	Calnexin	Polyclonal	# ab22595	1:1000 WB	Abcam
MAP2	Microtubule Associated Protein 2	Polyclonal	#ab92434	1:200 IF	Abcam

*WB, western blot; IHC, immunohistochemistry; IF, immunofluorescence.*

The cell samples were collected after treatment and fixed with 4% PFA for 20 min. Then, the cells were permeabilized with PBS containing 0.3% Triton X-100 for 20 min and blocked with 5% bovine serum albumin (BSA) for 30 min at room temperature. Without washing, the sections were incubated with the primary antibodies (Table 1) overnight, followed by incubation with a mixture of fluorescent secondary antibodies for 1 h at room temperature. The images were acquired using an intelligent inverted fluorescence microscope and confocal laser microscopy. The results of cell immunofluorescence experiments were independently repeated for three times. After staining, three independent fields were selected in each repeat experiment and the proportion of positive cells was counted for statistical analysis.

In order to quantify the complexity of the dendritic shape, MAP2 labeled neurons were reconstructed in Image J, and Sholl analysis was performed to calculate the intersection number (Ferreira et al., 2014). Five photos for statistical analysis in each group, and every photo has at least two neurons with complete morphology.

### Nissl Staining

The tissue sections were post-fixed with PFA for 15 min and washed with PBS. The sections were incubated in Nissl staining solution (Beyotime, China, Cat No. C0117) for 30 min at room temperature and washed with PBS. After color separation with alcohol and transparent with xylene, the sections were sealed with neutral balsam and observed in the microscope. Nissl’s bodies were calculated by Image J. Three mice were included in each group (Figure 8B, *N* = 3, Sham; Figure 8D, *N* = 3, intraluminal suture MCAO), calculate the number of positive staining cells in the same position of three different brain slices in each animal as the statistical parameter.

### TUNEL Staining

A one step TUNEL apoptosis assay kit (Beyotime, China, Cat No. C1086) was used to detect apoptosis according to the manufacturer’s instructions. Briefly, cells or tissues sections (four animals in each group)were cultured in confocal Petri dishes, rinsed with PBS and then fixed with 4% PFA for 15 min at room temperature. Next, the cells were washed in PBS for 5 min three times and subsequently incubated with 0.1% Triton X-100 in PBS for 2 min on ice. After that, the cells were washed in PBS three times and then incubated in TUNEL solution in the dark for 1 h at room temperature. Finally, the cells or tissues sections were washed and stained with DAPI (Beyotime, China, Cat No. C1005) or Hoechst (Beyotime, China, Cat No. C1017), followed by fluorescence microscopy imaging. Apoptosis levels were obtained by fluorescence positive/nuclear staining and were quantified by using ImageJ. The results of each TUNEL assay in cell experiments were independently repeated for three times and three animals in each group (Figure 8B, *N* = 3, Sham; Figure 8D, *N* = 3, intraluminal suture MCAO) were used. After staining, three independent fields were selected in each repeat experiment and the proportion of positive cells was counted for statistical analysis.

### Western Blotting

Cell proteins were obtained from harvested cells by western and IP cell lysis buffer (Beyotime, China, Cat No. P0013J) with 1% protease inhibitor cocktail (Millipore, United States, Cat No. 539131). After protein quantification by a BCA assay kit (Thermo Fisher, United States), 5–20 μg of protein were loaded onto 10 or 12% SDS-PAGE gels, and subsequently, the proteins were transferred to a 0.22 μm PVDF membrane (Millipore, United States). After the membrane was blocked with 5% milk, the membranes were incubated with the primary antibodies (Table 1) overnight at 4°C. After washing five times with TBST for 10 min, the membranes were incubated with secondary antibodies for 1 h at room temperature. After washing, the density of the protein bands was visualized and analyzed using a Tanon 2500 gel imaging system (Tanon, Shanghai, China). The western blot results for cell experiments were independently repeated for three times, and three animals in each group were used for this detection. All bands were quantified by ImageJ and normalized to β-actin, and the fold changes were calculated through relative quantification to control.

### Statistics

SPSS 13.0 software was used for all statistical analyses, and GraphPad Prism 8.0 software was used for graph generation. For western blot analysis, each set of data was derived from at least three independent experiments. For immunofluorescence and immunohistochemistry analyses, independent experiments were performed using 3–4 animals in each group and at least three sections of each brain tissue were used for analysis. Three different fields of view were randomly selected under a high-power microscope. The data are expressed as the mean and standard deviation. The difference in the mean in two groups was determined by independent sample *t*-tests, and if more than two groups were included, ANOVA was used. A value of *P* < 0.05 was considered statistically significant.

## Data Availability Statement

The raw data supporting the conclusions of this article will be made available by the authors, without undue reservation.

## Ethics Statement

The animal study was reviewed and approved by the Jinan University Laboratory Animal Ethics Committee.

## Author Contributions

All authors had full access to the manuscript and will take responsibility for the integrity of the data and the accuracy of the data analysis. AX and DL conceptualized and direct the overall project. JZ, YW, XS, XT, TZ, YuL, YaL, and XL performed the experiments. JZ, DL, CT, and DM wrote, edited, and proofread the manuscript.

## Conflict of Interest

The authors declare that the research was conducted in the absence of any commercial or financial relationships that could be construed as a potential conflict of interest.

## Abbreviations

3-MA: 3-methyladenine
MCAO: middle cerebral artery occlusion
NTA: nanoparticle tracking analysis
OGD: oxygen-glucose deprivation
PDE1-B: phosphodiesterase enzyme 1-B
TEM: transmission electron microscope.
