# Supplementary figures and images for: Inhibition of PDE1-B by Vinpocetine Regulates Microglial Exosomes and Polarization Through Enhancing Autophagic Flux for Neuroprotection Against Ischemic Stroke
Source: Front Cell Dev Biol. 2021 Feb 4;8:616590. doi: 10.3389/fcell.2020.616590 (PMC7889976; doi:10.3389/fcell.2020.616590)

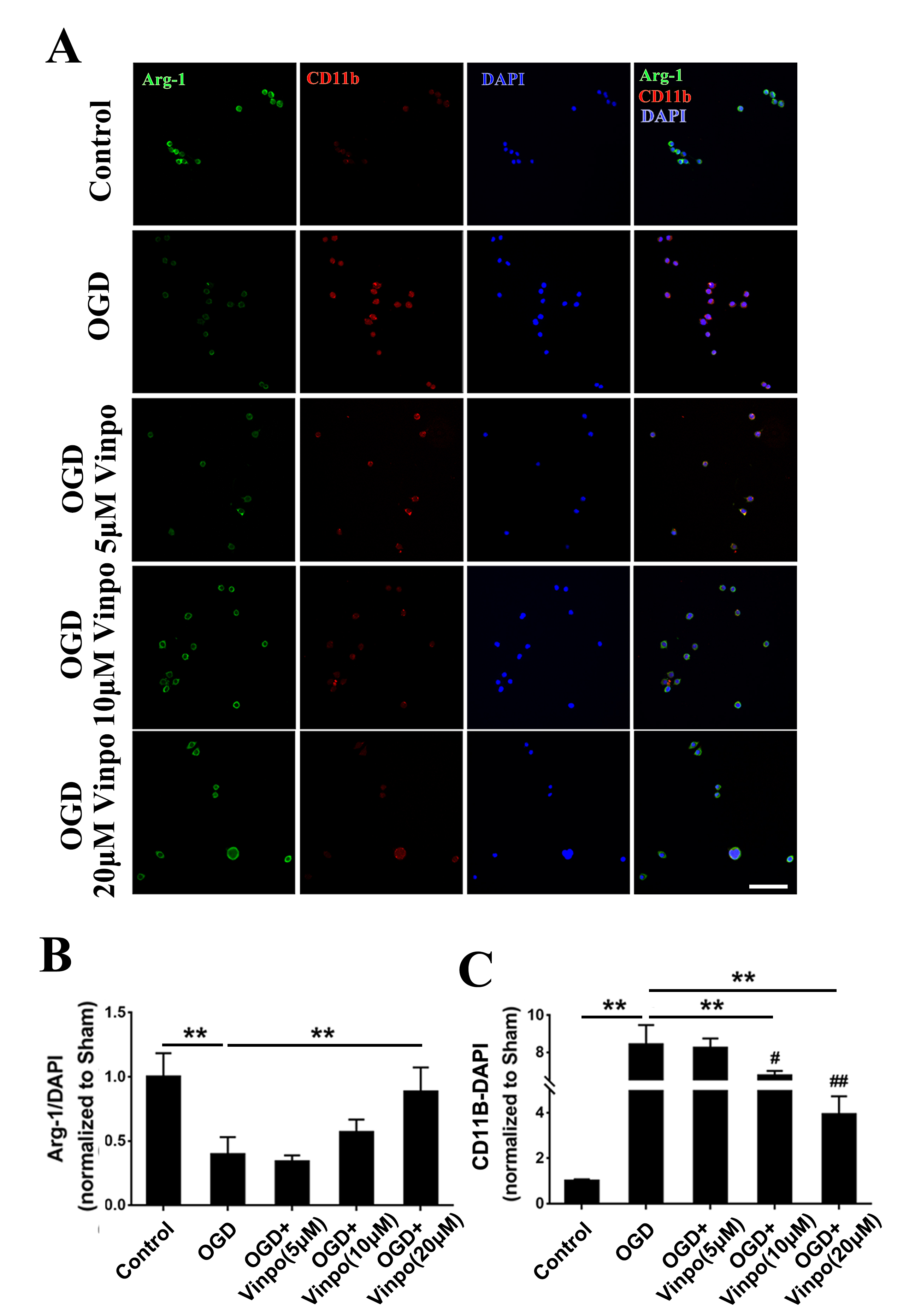

Supplement: Supplementary file 1 [file Image_1.JPEG]

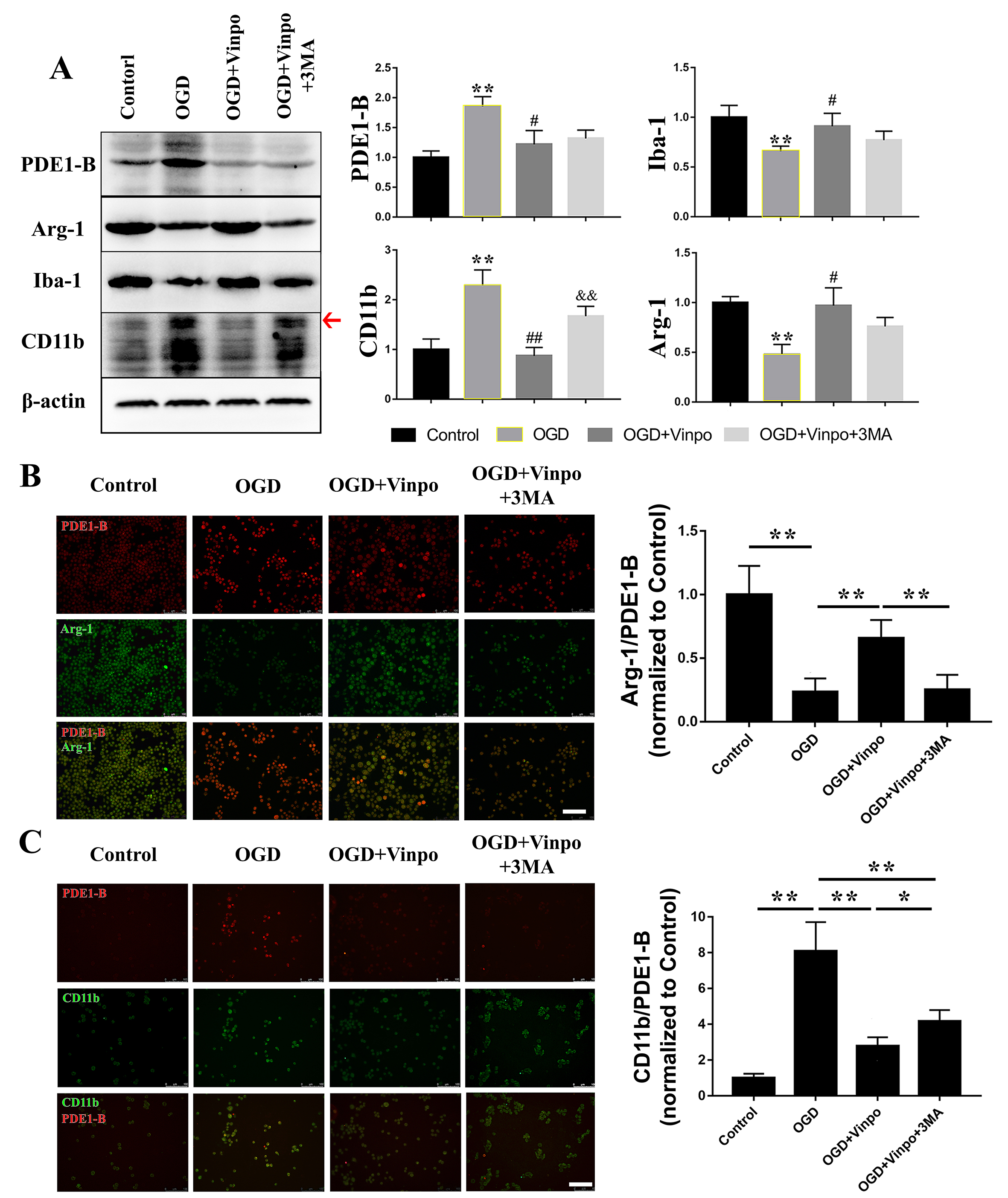

Supplement: Supplementary file 2 [file Image_2.JPEG]

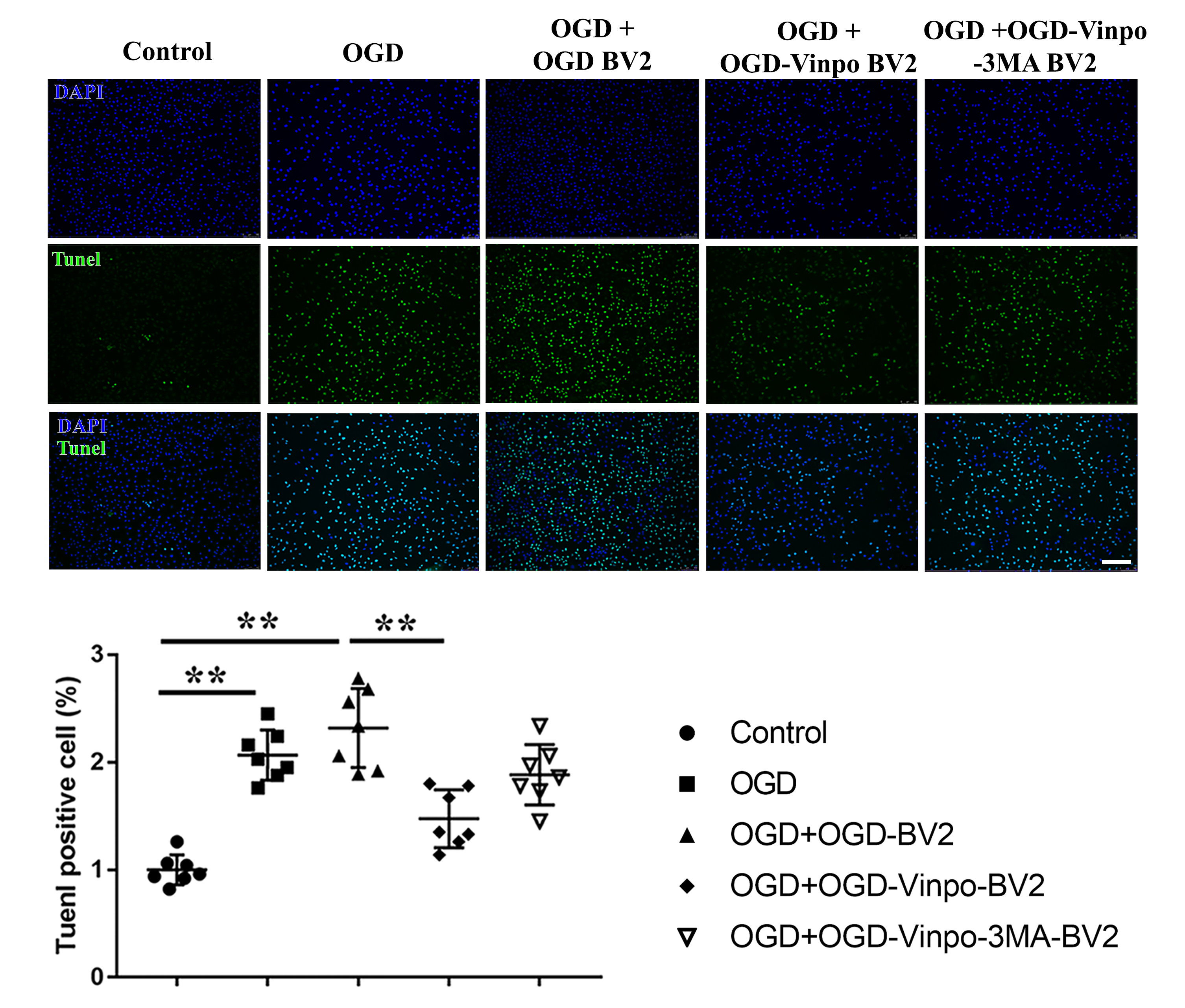

Supplement: Supplementary file 3 [file Image_3.JPEG]

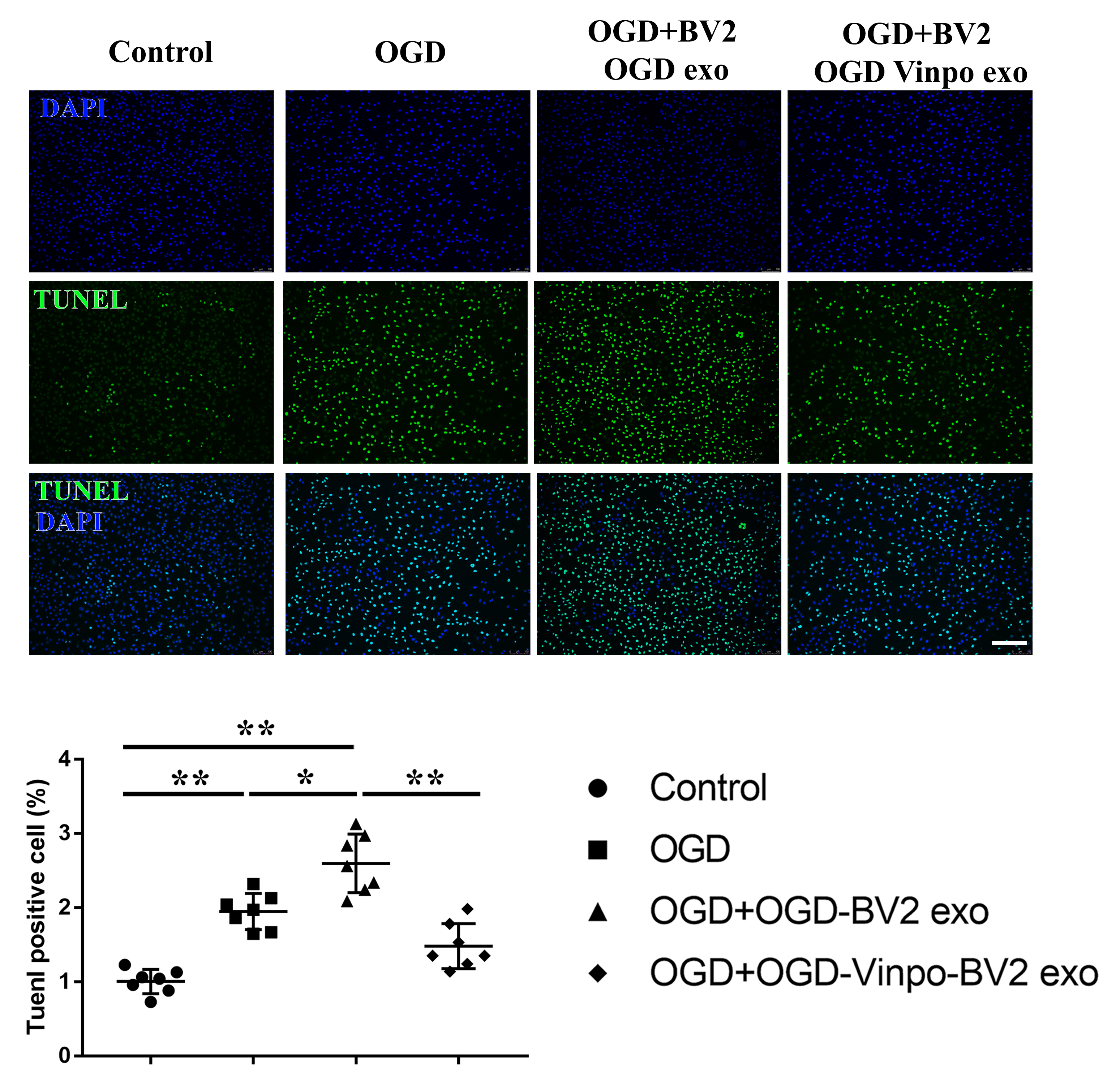

Supplement: Supplementary file 4 [file Image_4.JPEG]
