# Supplementary material for: Inhibition of PDE1-B by Vinpocetine Regulates Microglial Exosomes and Polarization Through Enhancing Autophagic Flux for Neuroprotection Against Ischemic Stroke
Source: Front Cell Dev Biol. 2021 Feb 4;8:616590. doi: 10.3389/fcell.2020.616590 (PMC7889976; doi:10.3389/fcell.2020.616590)
Supplement: Supplementary file 5 [file Table_1.DOCX]

**Figure S1. Vinpocetine treatment inhibits BV2 M1 and promotes M2** **phenotype in OGD condition.**

**(A)** Representative immunofluorescence images of Arg-1 and CD11b positive BV2 cells pre-treated with the indicated concentrations of vinpocetine for 24 hours before OGD incubation for 3 hours or in normal medium (control). **(B-C)** Quantification of Arg-1 and CD11b positive cells in (A). **p < 0.01. Scale bar=100 μm.

**Figure S2. Blocking autophagy respond inhibits vinpocetine-induced BV2 M1 phenotype reduce and M2 activation.**

**(A)** Representative immunoblots and quantification of PDE1-B, Arg-1, Iba-1, CD11b and β-actin in BV2 cells treated with vinpocetine (Vinpo) or/and 3-MA for 24 hours after OGD incubation for 3 hours or in normal medium (control). Whole cell lysates were used for immunoblotting. **p < 0.01 versus control; ^#^p < 0.05 and ^##^p < 0.01 versus OGD; ^&&^p < 0.01 versus OGD+vinpo. **(B-C)** Representative immunofluorescence images of CD11b and Arg-1 co-staining with PDE1-B in BV2 cells treated as described in (A). *p < 0.05 and **p < 0.01, Scale bar=100 μm.

**Figure S3. Vinpocetine-treated OGD-conditioned BV2 cells alleviates OGD-induced SH-SY5Y apoptosis**

Representative immunofluorescence images of TUNEL staining in SY5Y cells co-cultured with BV2 cells treated as described in Fig. 3A and Method section. Scale bar=100 μm.

**Figure S4. The exosomes derived from vinpocetine-treated OGD-conditioned BV2 cells ameliorates OGD-induced SH-SY5Y apoptosis**

Representative immunofluorescence images of TUNEL staining in SY5Y cells were incubated with the purified exosomes isolated from different conditioned BV2 cells treated as described in Fig. 3A. Scale bar=100 μm.
